# Commentary on ‘Evaluating outcomes of primary anastomosis versus Hartmann's procedure in sigmoid volvulus: A retrospective-cohort study’

**DOI:** 10.1016/j.amsu.2021.02.018

**Published:** 2021-02-12

**Authors:** Sabri Selcuk Atamanalp

**Affiliations:** Department of General Surgery, Faculty of Medicine, Ataturk University, 25040, Erzurum, Turkey

Dear editor,

I read the article by Shahmoradi et al. [1] regarding the outcomes of surgical treatment in sigmoid volvulus (SV). SV, the twisting of the sigmoid colon around its own mesentery causing an intestinal obstruction, is quite rare worldwide [2], while it is relatively common in Turkey, particularly in Eastern Anatolia, my practicing area [3]. Under favour of our experience on SV including 1036 cases over a 54.5-year period (between June 1966 and January 2021), which is the largest single-center SV series over the world [4], I want to discuss some details of the emergency surgical treatment of SV regarding the presentation of the authors.

First, although the present study outlines the outcomes of primary anastomosis and Hartmann's procedure following sigmoid resection in SV, the retrospective nature of the study is a limitation in a thorough evaluation. It is clear that, to obtain a completely randomized grouping is not easy in a perennial retrospective study. As a common clinical practice, well-conditioned patients are generally treated with primary anastomosis, while stoma, which is an undesired but a life saver procedure when needed, is commonly preferred in bed-conditioned cases in the emergency surgical treatment of SV [5,6], which is also my personal preference and suggestion.

Second, the distribution of some preoperative parameters or related statistical analyses are not clear enough in the authors’ article. Among these, although the American Society of Anesthesiologists (ASA) score is given in total, its distribution according to the groups and the statistical analysis are not found. Similarly, although the distribution of comorbid diseases and bowel gangrene are documented in both groups, their statistical analyses are not offered. Although some postoperative parameters including mortality, morbidity, and hospitalization period demonstrate statistically similar results in both groups, their postoperative evaluation without the statistical comparison of the preoperative data may open the credibility of the results up for discussion. It is clear that, patients with worse preoperative parameters may reveal worse prognostic data [7]. In my opinion, if possible, to perform a detailed statistical analysis, particularly a propensity score matching, may give more meaningful results in the present series.

Last, regarding the mortality rate of the authors' series, a 1,0% of total mortality rate (0.0% in primary anastomosis group and 1.8% in Hartmann's procedure group) is an excellent result, particularly in patients with 23.1% of sigmoid gangrene and 9.6% of perforation. In our surgically treated 478-patient series, overall mortality rate is 16.9%, while it is 23.5% (67/285) in patients with sigmoid gangrene and 47.1% (8/17) in cases with perforation. Despite advanced pre-, per- and postoperative techniques and care, emergency SV surgery has still a relatively poor prognosis with a 1–40% of mortality rate in patients with viable bowel, while this rate may rise 5–60% in gangrenous patients [6,7].

I congratulate the authors for their paper and look forward to their reply on my comments.

## Ethical approval

No ethical approval required.

## Source of funding

No funding.

## Author contribution

Sabri Selcuk Atamanalp designed the study, searched the literature, wrote the text, evaluated the data, approved the final form.

## Trial registry number

Name of the registry:

Unique Identifying number or registration:

Hyperlink to your specific registration (must be publicly accessible and will be checked):

## Guarantor

Sabri Selcuk Atamanalp.

## Declaration of competing interest

The author declares that he has no conflict of interest.
